# Cardiac Molecular-Acclimation Mechanisms in Response to Swimming-Induced Exercise in Atlantic Salmon

**DOI:** 10.1371/journal.pone.0055056

**Published:** 2013-01-25

**Authors:** Vicente Castro, Barbara Grisdale-Helland, Ståle J. Helland, Jacob Torgersen, Torstein Kristensen, Guy Claireaux, Anthony P. Farrell, Harald Takle

**Affiliations:** 1 Nofima AS, Ås, Norway; 2 Institute of Animal Sciences, Norwegian University of Life Sciences (UMB), Ås, Norway; 3 Aquaculture Protein Centre, Ås, Norway; 4 Nofima AS, Sunndalsøra, Norway; 5 Norwegian Institute of Water Research, Bodø, Norway; 6 Laboratoire des Sciences de ĺEnvironnement Marin (LEMAR), Université de Bretagne Occidentale, & Unité de Physiologie Fonctionnelle des Organismes Marins, Ifremer, Plouzané, France; 7 Faculty of Land and Food Systems, & Department of Zoology, University of British Columbia, Vancouver, British Columbia, Canada; 8 AVS Chile SA, Puerto Varas, Chile; Universitat de Barcelona, Spain

## Abstract

Cardiac muscle is a principal target organ for exercise-induced acclimation mechanisms in fish and mammals, given that sustained aerobic exercise training improves cardiac output. Yet, the molecular mechanisms underlying such cardiac acclimation have been scarcely investigated in teleosts. Consequently, we studied mechanisms related to cardiac growth, contractility, vascularization, energy metabolism and myokine production in Atlantic salmon pre-smolts resulting from 10 weeks exercise-training at three different swimming intensities: 0.32 (control), 0.65 (medium intensity) and 1.31 (high intensity) body lengths s^−1^. Cardiac responses were characterized using growth, immunofluorescence and qPCR analysis of a large number of target genes encoding proteins with significant and well-characterized function. The overall stimulatory effect of exercise on cardiac muscle was dependent on training intensity, with changes elicited by high intensity training being of greater magnitude than either medium intensity or control. Higher protein levels of PCNA were indicative of cardiac growth being driven by cardiomyocyte hyperplasia, while elevated cardiac mRNA levels of MEF2C, GATA4 and ACTA1 suggested cardiomyocyte hypertrophy. In addition, up-regulation of EC coupling-related genes suggested that exercised hearts may have improved contractile function, while higher mRNA levels of EPO and VEGF were suggestive of a more efficient oxygen supply network. Furthermore, higher mRNA levels of PPARα, PGC1α and CPT1 all suggested a higher capacity for lipid oxidation, which along with a significant enlargement of mitochondrial size in cardiac myocytes of the compact layer of fish exercised at high intensity, suggested an enhanced energetic support system. Training also elevated transcription of a set of myokines and other gene products related to the inflammatory process, such as TNFα, NFκB, COX2, IL1RA and TNF decoy receptor. This study provides the first characterization of the underlying molecular acclimation mechanisms in the heart of exercise-trained fish, which resemble those reported for mammalian physiological cardiac growth.

## Introduction

Aerobic exercise training imposes an intensity-dependent cardiac load in vertebrates in response to an increased need for internal oxygen transport. The cardiac growth in aerobically trained humans is typically associated with improved physical capacity [1]–[3]. Conversely, pathological cardiac growth occurs in response to volume overload in disease settings (e.g. hypertension), myocardial infarction and ischemia [4]. The fish cardiovascular system is similarly an important target for the training-induced effects, being plastic [5] and modulated by training intensity [6], [7]. Cardiovascular improvements in fish include maximum oxygen consumption [7], maximum cardiac output [8], [9], tissue capillarization [10], [11], oxygen extraction by tissues [12], haematocrit [7], [13], [14], tissue oxidative capacity [8] and relative cardiac size [5], [15]. Clearly, exercise training in fish targets many same levels of the oxygen cascade as it does in mammals.

Cardiac mass, on which cardiac stroke volume depends, is highly plastic to environmental and physiological stressors in fish, e.g. sexual maturation, cold temperature acclimation and anemia [5], [16]–[18]. For example, sexual maturation [19], [20] and anemia conditions [21] triggered both cardiomyocyte enlargement (hypertrophy) and proliferation (hyperplasia), while exercise training triggered cardiac hyperplasia, as evidenced by elevated transcription of proliferating cell nuclear antigen (PCNA) in zebrafish (*Danio rerio*) [22]. In contrast, mammalian cardiomyocytes practically lose their ability to proliferate after birth, growing mainly through cellular hypertrophy [23] governed by the expression of transcription factors such as myocyte enhancer factor (MEF)2C, GATA4 and the homeobox protein NKX2.5 [24]. Thus, the cellular responses of fish and mammals to environmental stressors can differ considerably.

Exercise training in mammals elevates transcription of cardiac genes encoding proteins involved in the excitation–contraction (EC) coupling process and in the handling of Ca^2+^ fluxes required for contraction activation such as the voltage dependent L-type Ca^2+^ channel (dihydropyridine receptor; DHPR), the sarcoplasmic reticulum (SR) Ca^2+^ release channel (ryanodine receptor; RYR) and the SR Ca^2+^ ATPase (SERCA), responses that likely improve the contractile function of the myocytes [25]–[27]. However, to our knowledge, no information is published on the exercise training regulation of these molecules in cardiac muscle of fish. Nevertheless, there is good reason that certain proteins may be targeted. For example, an association exists between DHPR or RYR and skeletal muscle contractile performance [28]–[30]. Exercise training in fish improves capillarity, favouring oxygen diffusion to mitochondria in skeletal muscles [7], [10], [31], [32], and stimulates erythropoiesis, as evidenced by increased haematocrit levels [14]. Such training effects are likely driven by vascular endothelial growth factor (VEGF), the most important angiogenic factor in vertebrates [33], and the principal regulator of erythropoiesis, erythropoietin (EPO), which was recently cloned in fish and was found to be mainly produced in the heart and not in the kidney, as for mammals [34].

Cardiac muscle of high performance fish such as tunas and salmonids, like humans, prefers lipids as a fuel under normal conditions [35], [36]. Further, lipid use increases during aerobic swimming while the use of glucose remains limited [37]. Peroxisome proliferator activated receptor (PPAR)α is a key cardiac transcription factor regulating lipid catabolism pathways by inducing the transcription of genes such as *carnitine palmitoyltransferase 1* (*CPT1*) [38]. PPARγ co-activator (PGC)1α is a cardiac-enriched PPAR coactivator that directly activates PPARα, boosting its effects at the same time of co-activating other transcription factors involved in mitochondrial biogenesis. Exercise-induced myokine regulation, a set of cytokines, may be another important molecular acclimation mechanism affecting cardiac performance because regular exercise training in mammals induces skeletal muscle myokine production and release [39]. A modulation in cardiac myokines production in response to training was recently shown for Atlantic salmon (*Salmo salar*) [40].

In view of the above, we hypothesized that the many pathways involved in strengthening the cardiovascular capacity are conserved among fishes and terrestrial vertebrates. Further, we hypothesized that exercise-induced activation of underlying gene transcription mechanisms must be dependent on the cardiac workload. To test our hypotheses, we trained Atlantic salmon pre-smolts at three different exercise intensities for 10 weeks and then analyzed key markers of pathways affecting the traits known to be involved in cardiomyocyte growth and proliferation, contractility, capillarization, oxygen transport, myokine production, energy metabolism and fuel preference.

## Results

### Cardiac Growth and Contractile Capacity

After 10 weeks of training, average mass and length of sampled fish were similar: 99.6±5.7 g and 19.9±0.33 cm for the control (C); 91.7±1.7 g and 19.2±0.16 cm for the medium intensity (M), and 92.8±5.2 g and 19.6±0.13 cm for the high intensity (H) groups, respectively (p>0.05). Yet, ventricular mass relative to body mass (RVM) of trained fish had grown more than control fish by 11.1% for M and 19.4% for H: RVM values were 0.087±0.004%; 0.097±0.007% and 0.104±0.007% for C, M and H, respectively. Hence, a clear tendency (p = 0.06, Student’s t-test) existed for cardiac growth to be intensity-dependent between C and H.

**Figure 1:**
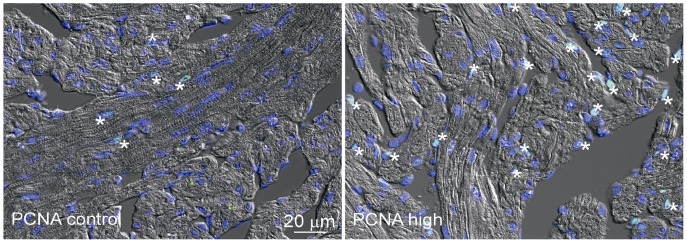
Exercise training and cardiomyocyte proliferation. Immunofluorescence detection of PCNA (green) in spongy myocardium. Control fish showed modest cell proliferation (*) with an average of three positive cells per frame (left image). PCNA staining of exercised fish from the high intensity-training regime (right image) shows a considerable increase (20 positive cells per frame) in cell proliferation over specimen from the Control group. Nuclei are stained with DAPI (blue). n = 12/group.

**Figure 2:**
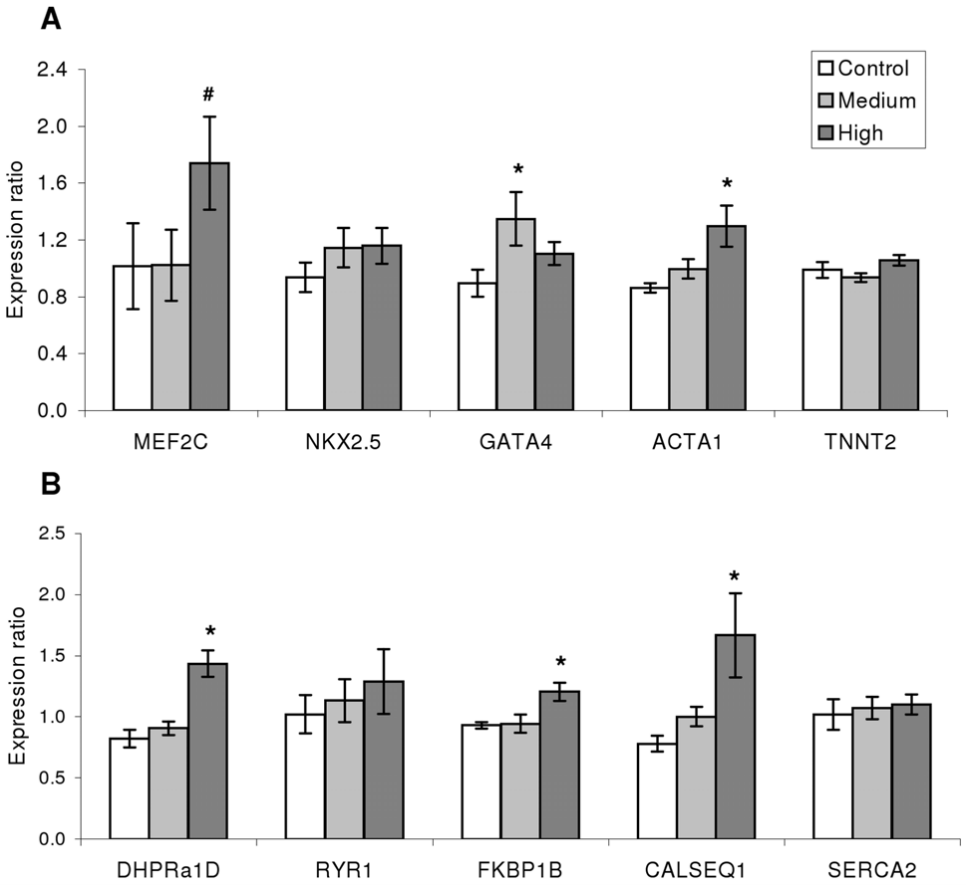
Cardiomyocyte growth and contractile capacity are affected by exercise. Gene expression related to the growth and contractile capacity of Atlantic salmon cardiomyocytes was analyzed by qPCR. A: Genes related to cardiomyocyte growth. B: Genes involved in the EC-coupling process as well as in Ca^2+^ handling. * denotes statistical difference (p<0.05; one-way ANOVA performed on log2 transformed expression ratio values followed by Tukey’s HSD; n = 9−12/group) between either of the training regimes and the Control. # denotes significant difference between C and H (Students *t*-test; p<0.05). Bars represent SEM. MEF2C: Myocyte-specific enhancer factor 2C; GATA4: GATA binding protein 4; NKX2: NK2 homeobox 5; ACTA1: Actin; TNNT2: Troponin; DHPRa1D: Voltage dependent L-type Ca^2+^ channel alpha1D subunit (dihydropyridine receptor); RYR1: Sarcoplasmic reticulum Ca^2+^ release channel (ryanodine receptor) isoform1; FKBP1B: FK506 binding protein B; CALSEQ1: Calsequestrin 1; SERCA2: Sarcoplasmic reticulum Ca^2+^ ATPase 2.

To evaluate hyperplastic cardiac growth, ventricular myocyte proliferation was assessed by PCNA immunofluorescence and found to be ∼7 fold greater in H relative to C (p = 0.05; Figure 1). Expression of ten genes directly related with processes of cardiac muscle growth, development, contraction machinery and EC coupling was assessed by qPCR to further define the molecular signature behind cardiac growth (Figure 2). The transcription levels of these markers tended to be higher in trained than control fish, suggesting hypertrophic cardiomyocyte growth had occurred as well (Figure 2A). The H regime triggered significant differences for the genes encoding MEF2C and actin alpha 1 (ACTA1) when compared to C. Conversely, the M regime triggered only up-regulated levels of GATA4 in comparison to C. Similarly, key marker genes associated with the contractile machinery, specifically those encoding DHPR, FK-506 binding protein (FKBP1B) and calsequestrin 1 (CALSEQ1), showed significantly higher transcription with the H regime compared to C (Figure 2B).

### Capillarization and Oxygen Carrying Capacity

**Figure 3:**
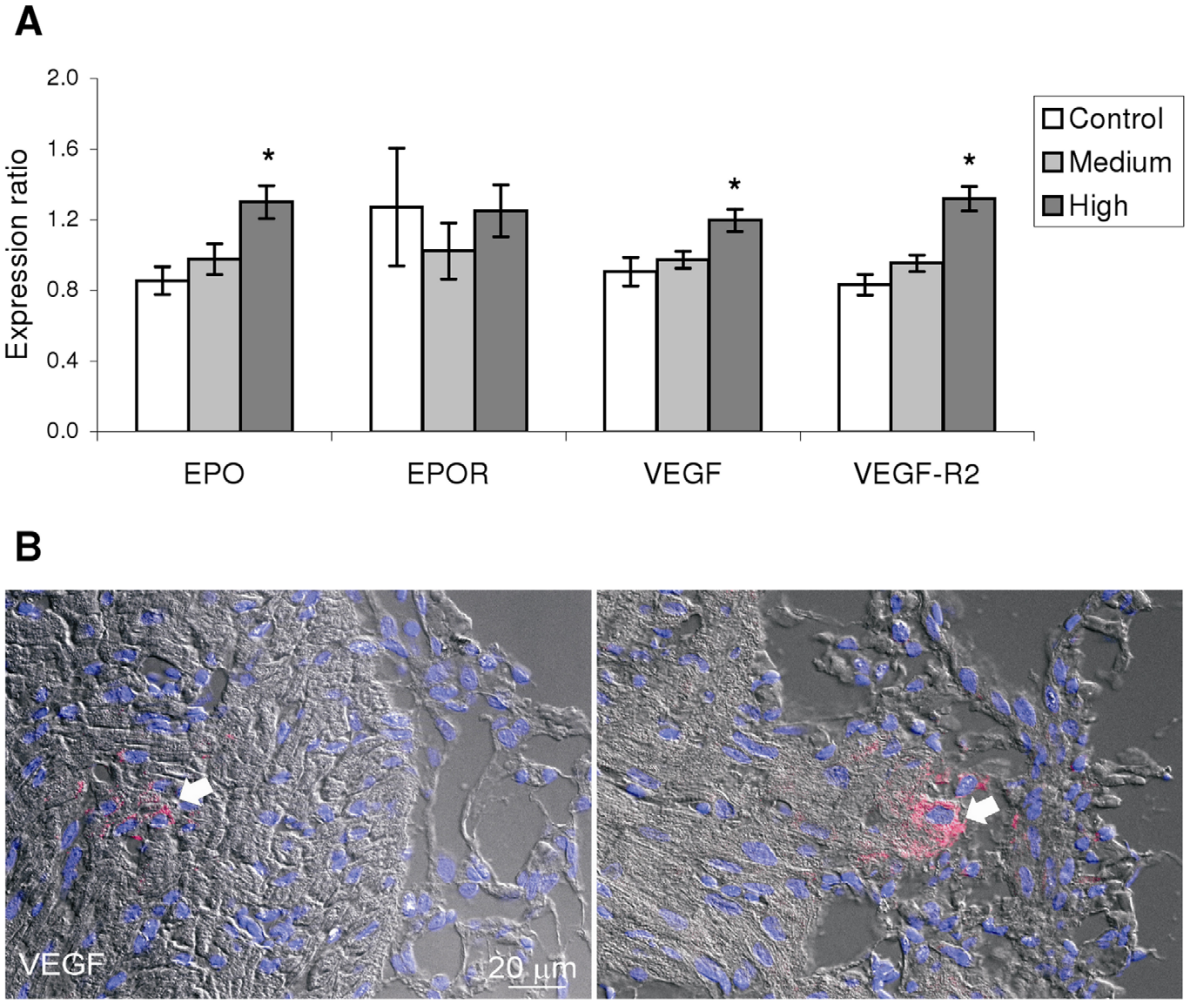
Oxygen carrying and distribution capacity is affected by exercise training. A: High intensity exercise training stimulated up-regulation of genes coding for EPO, involved in erythropoiesis, and for VEGF and VEGF receptor (VEGF-R2), which mediates angiogenesis. B: Immunofluorescence detection image of VEGF (arrows, red staining) showing its production in both the epicardium (right image) and compact myocardium (left image) suggesting the formation of new blood vessels. No apparent differences were found between high intensity trained and control fish (nuclei: blue (DAPI)). * denotes statistical difference (p<0.05; one-way ANOVA performed on log2 transformed expression ratio values followed by Tukey’s HSD; n = 9−12/group ) between either of the training regimes and the Control. Bars represent SEM. ER: Expression ratio. EPO: erythropoietin; EPOR: EPO receptor; VEGF: Vascular endothelial growth factor; VEGF-R2: VEGF receptor 2.

Improvements to oxygen carrying capacity and the coronary vasculature would provide better cardiac oxygen and nutrient delivery. Five genes involved in oxygen transport and blood diffusion were studied by qPCR (Figure 3A; *inducible nitric oxide synthase* –*iNOS*- was undetectable). Transcription levels of *EPO* were significantly higher in ventricle of H-trained fish, while the *EPO receptor* (*EPOR*) was unchanged. Training significantly increased transcription levels of *VEGF* and its cell surface receptor *VEGF-R2* with the H regime compared to C. The spatial expression of VEGF was localized in the ventricular epi- and myocardium for both H and C regimes, while a difference in protein levels was not found by IF (Figure 3B).

### Energy Metabolism

**Figure 4:**
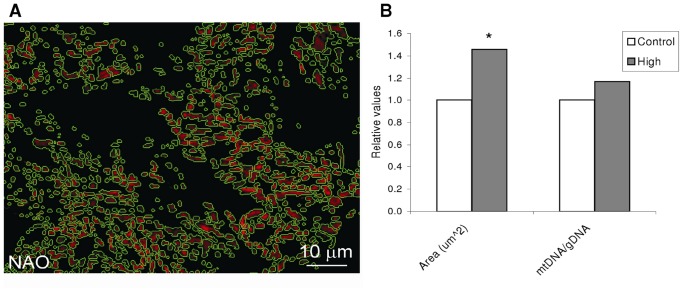
Exercise effects on mitochondrial size and density. A: Fluorescence microscopy of Acridine orange 10-nonyl bromide (NAO) stained mitochondria (pseudo coloured red) in spongy myocardium. The segmentation masks are shown as green lines around the mitochondria. B: Quantification of fluorescence intensity inside the segmentation masks showed that mitochondrial area within the cardiomyocytes of Atlantic salmon was significantly increased by the high-intensity training regime (approx. 16000 mitochondrial from sections of 5 and 6 hearts from control and high-intensity regimes, respectively). The number of mitochondria per cell though, was not significantly different as shown by that the ratio between mitochondrial DNA and genomic DNA (mtDNA/gDNA). *: Student’s *t*-test p<0.05, performed on metric and log2ER values for area and mtDNA/gDNA respectively. n = 12/group.

A greater cardiac oxidative capacity was indicated by the mitochondria area being significantly larger (46%) in H compared with C (1.23±0.015 µm^2^; n = 15620 and 0.84±0.09 µm^2^; n = 17780, respectively). The mitochondrial to genomic DNA ratio (mtDNA/gDNA) was unaffected by exercise (Figure 4).

**Figure 5:**
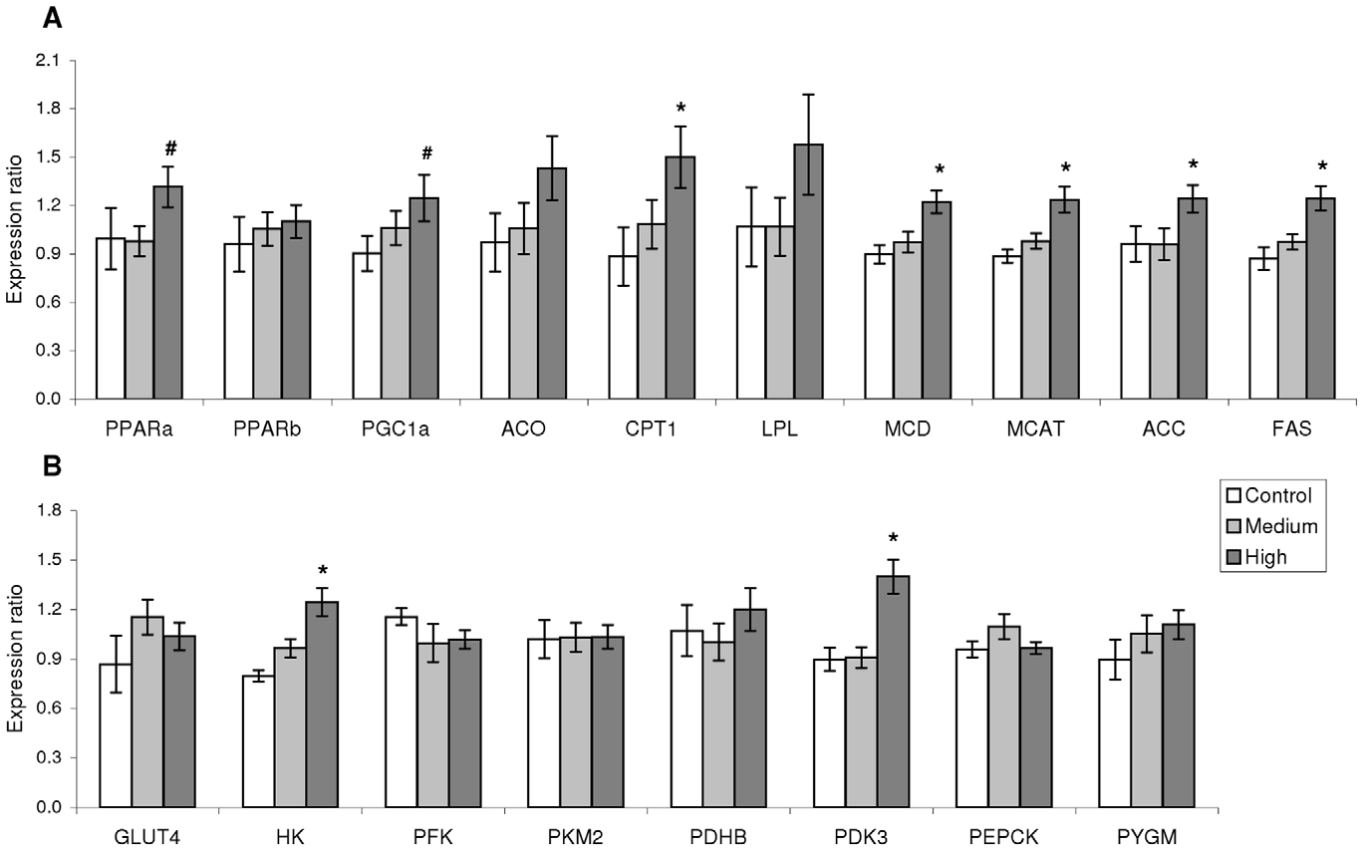
Exercise affects cardiac metabolism. A: A strong trend towards up-regulation of genes participating in the metabolism of lipids was seen only for the high-intensity regime compared with control, while the metabolism of carbohydrates (B) was not necessarily affected. * denotes statistical difference (p<0.05; one-way ANOVA performed on log2 transformed expression ratio values followed by Tukey’s HSD; n = 9−12/group) between either of the training regimes and the Control. # denotes significant difference between C and H (Students *t*-test; p<0.05). Bars represent SEM. ER: Expression ratio. PPARa: Peroxisome proliferator activated receptor (PPAR)α; PPARb: PPARβ; PGC1a: PPARγ coactivator 1α; ACO: Acyl-CoA oxidase; CPT1: Carnitine palmitoyltransferase 1; LPL: Lipoprotein lipase; MCD: Malonyl-CoA carboxylase; MCAT: Malonyl-CoA acyl carrier protein transacylase; ACC: Acetyl-CoA carboxylase; FAS: Fatty acid synthase; GLUT4: Glucose transporter type 4; HK: Hexokinase; PFK: Phosphofructokinase; PKM2: Pyruvate kinase isoform 2; PDHB: Pyruvate dehydrogenase E1 component subunit β; PDK3: Pyruvate dehydrogenase kinase 3; PEPCK: phosphoenolpyruvatecarboxykinase; PYGM: Glycogen phosphorylase.

Insight into cardiac fuel preference was assessed using qPCR expression of 18 genes involved in the metabolism of lipids and glucose. Exercise training had a profound effect on the expression of those genes involved in lipid metabolism since 7 out of 10 genes were consistently up-regulated in H compared with C (Figure 5A). These genes included those encoding proteins promoting mitochondrial biogenesis and fatty acid oxidation such as PPARα, PGC1α, CPT1, and malonyl-CoA decarboxylase (MCD). Furthermore, mRNA of proteins participating in lipogenetic pathways were also up-regulated by H training, including malonyl CoA-acyl carrier protein transacylase (MCAT), acetyl CoA carboxylase (ACC) and fatty acid synthase (FAS). In addition, two (*hexokinase* (*HK*) and *pyruvate dehydrogenase kinase* (*PDK3*)) out of eight genes involved in glucose metabolism were significantly up-regulated in the H regime compared to C (Figure 5B). In contrast, none of these genes were differentially expressed between M and C.

### Cardiomyokine Expression

**Figure 6:**
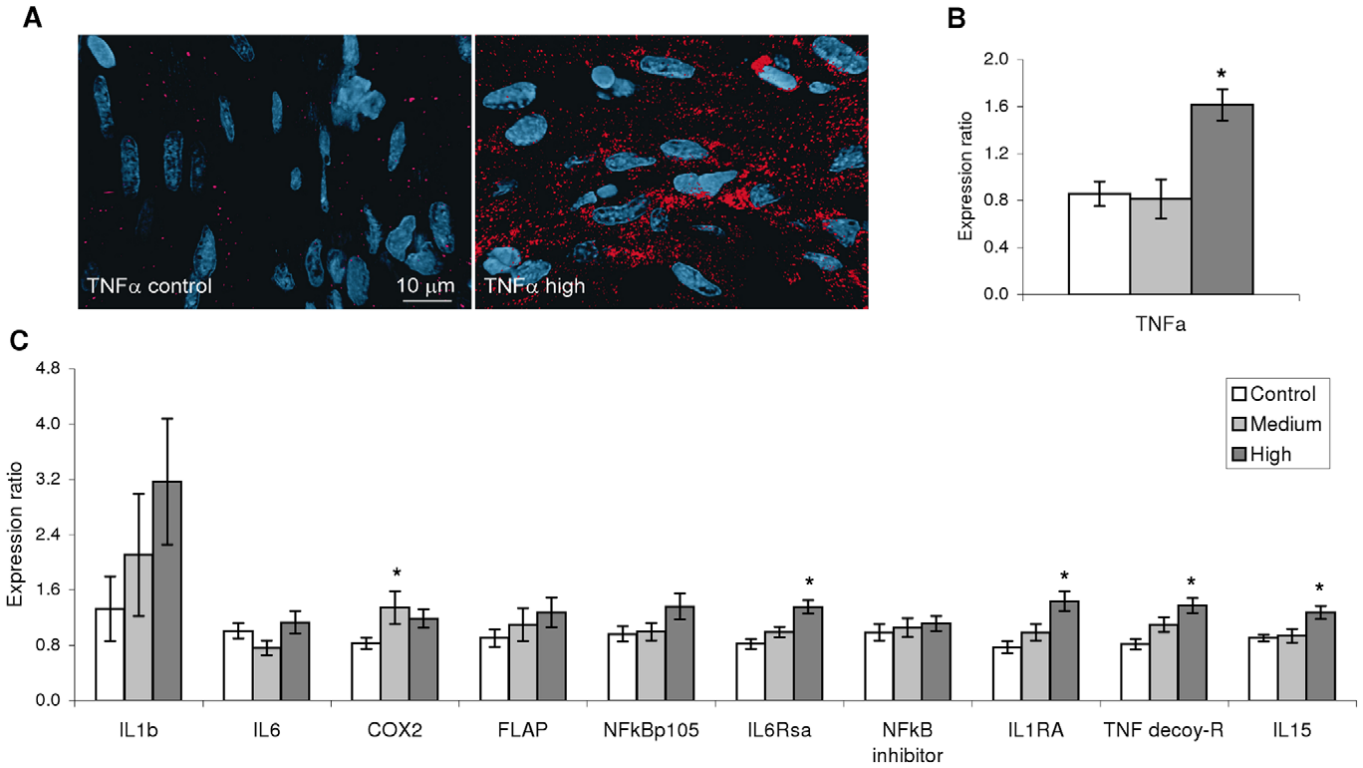
Cardiac inflammatory response to exercise training. Both protein (A) and transcript (B) levels of TNFα were significantly upregulated by the high-intensity training regime. A: Immunofluorescence detection of TNFα (red) in cardiomyocytes of Control (left picture) and High intensity (right) shows how TNFα is strongly induced by exercise training in the compact myocardium of Atlantic salmon (n = 12/regime). Nuclei were labelled with DAPI (blue). B: qPCR revealed mRNA levels of TNFα being up-regulated by the high-intensity regime compared with control. C: Expression of a set of genes associated with both pro- and anti-inflammatory mechanisms was consistently induced by the High intensity regime, while expression of only two genes was significantly stimulated by Medium intensity training in relation to Control. * denotes statistical difference (p<0.05; one-way ANOVA performed on log2 transformed expression ratio values followed by Tukey’s HSD; n = 9−12/group). Bars represent SEM. ER: Expression ratio. IL1b: Interleukin 1β; IL6: Interleukin 6; COX2: Cyclooxygenase 2; FLAP: 5-lipoxygenase-activating protein; NFkBp105: Nuclear factor κBp105; NFκB inhibitor: Nuclear factor κB inhibitor α1; IL6Rsa: IL6 receptor subunit α; IL1RA: IL1 receptor antagonist; TNF decoy-R (receptor); IL15: Interleukin 15.

Aerobic exercise increased cardiac expression of pro- and anti-inflammatory cytokines in an intensity-dependent manner. Protein and mRNA levels of tumor necrosis factor (TNF)α were significantly up-regulated in H compared with C, as shown by IF and qPCR, respectively (Figures 6A & 6B). Further, IF analysis allocated TNFα production to the cardiomyocytes and not to potential resident leukocytes. Another significantly up-regulated pro-inflammatory-related gene in H was the *interleukin (IL)6 receptor subunit alpha* (*IL6Rsα*), while M triggered elevated transcription of *cyclooxygenase* (*COX*)*2*. Within the genes with anti-inflammatory properties, H significantly induced the transcription of *TNF decoy* receptor and *IL1 receptor antagonist* (*IL1RA*). Further, *IL15*, which has been linked to muscle growth [41], was up-regulated by H (Figure 6C).

### Exercise Intensity

Overall, intensity of the training regime was directly related to the magnitude in the gene expression response (Pearson correlation; *r* = 0.99; p = 0.032). Furthermore, significant differences were found between the three groups (p<0.0001), with M and H displaying a 10.9% and 37.8% higher gene expression than C, respectively.

## Discussion

In this study, we demonstrate that genes and proteins known to be key components of the molecular signature for mammalian cardiac growth stimulated by exercise also underlie the exercise-induced responses of the Atlantic salmon heart.

Training intensity influenced cardiac growth and gene expression, with lower values in C, a low-to-moderate effect in M and higher magnitude in H. While intense training has previously triggered cardiac improvements in mammals and fish, those that are not sufficiently intense or too short may not elicit cardiovascular changes [7], [42], [43]. Optimal effects of exercise training in salmonids, including growth, reduced stress and disease resistance, are apparently achieved at swimming speeds between 1 and 1.5 body lengths (BL) s^−1^ [15], [40], [44], and so our significant effects with the H-training regime (1.31 BL s^−1^) are in line with previous studies, and add a molecular dimension to the training effects.

Fish robustness is thought to be enhanced by a relatively larger cardiac mass, perhaps improving cardiovascular capacity to satisfy the demands for simultaneous swimming and growth. Indeed, better swimmers and wild fish have a larger RVM. Here, the 19.4% RVM increment for the H regime is generally higher than in previous reports for exercise training in salmonids [7], [13], [45], though studies have found the opposite result [46]. We suggest that training-induced cardiac enlargement involved a mixture of hyperplasia and hypertrophy, given the higher protein levels of PCNA and the higher transcript amounts of the morphogenic- and hypertrophic-related genes *MEF2C*, *GATA4* and *ACTA1*. Cardiac growth in salmonids can also occur via both above-mentioned mechanisms in response to anemia or sexual maturation [19]–[21]. We did not attempt to resolve sexual dichotomy in training cardiac effects, but such differences should not be excluded. However, an enlarged heart working harder during exercise necessarily requires an improved contractile and metabolic capacity. Several observations were consistent with such thinking.

In mammals, improved contractile capacity is an important associated feature of physiological cardiac growth and is reflected by higher expression of Ca^2+^ channels and a better Ca^2+^ homeostasis [4], [47]. Similarly, the H regime triggered higher transcription activity of genes involved in Ca^2+^ handling during EC coupling. Indeed, increased transcription of *FKBP1B* and *CALSEQ1* suggested higher reliance on SR-stored Ca^2+^ to support the higher cardiac contractility in the greater swimming activity of the H regime. In cardiac muscle, FKBP1B is a RYR modulator, while CALSEQ1 is the most important Ca^2+^ binding protein inside the SR [48]. While increased reliance on SR-Ca^2+^ has been previously shown for cold acclimation in salmonids [49]–[51], the present work is the first to suggest a similar response in response to exercise training. It must be noted that these results may also be reflective of an increased protein turnover due to exercise-induced cellular damage or increased activity.

The working myocardium needs oxygen and the coronary circulation to the compact layer of ventricle in salmon ensures delivery of freshly oxygenated blood to the working heart during intensive exercise [8]. The up-regulated mRNA levels of both VEGF and its receptor between H and C suggests increased cardiac capillarization with exercise training, especially since VEGF protein expression was localized to the myocardium eventhough IF did not resolve differences in VEGF expression. In mammals, exercise-induced VEGF and VEGF-R mRNA and protein levels are associated with amelioration of the ageing-related decrease in cardiac capillarization and blood supply [52]–[54]. Furthermore, higher levels of EPO mRNA in the H-trained group suggest an increased hematopoietic capacity, which in turn suggests a molecular mechanism underlying previously observed increases in haematocrit, haemoglobin concentration and blood oxygen carrying capacity in exercise-trained teleosts [15]. Similarly, EPO production is induced in response to exercise training in mammals, and is further associated with increased lipid metabolism, muscle hypertrophy and capillarization [55]–[58].

Larger cardiac mitochondrial size of H-trained fish further suggested improved respiratory capacity, while higher transcript abundance of PPARα, PGC1α, CPT1 and MCD suggests greater cardiac reliance on lipids oxidation for energy generation. PPARα and PGC1α are key drivers of mitochondrial biogenesis and β-oxidation [59], [60], while PGC1α appears to be a key mediator of several of the beneficial effects of exercise in mammals [61]. Conversely, PPARα and PGC1α transcription is repressed in pathological mammalian cardiac growth [38]. Increased mRNA expression of CPT1, whose transcription is stimulated by PPARα, indicates higher transport of long-chain fatty acids into the mitochondria [59]. MCD converts malonyl-CoA into acetyl-CoA, thereby reducing the inhibitory effects that malonyl-CoA imposes on β-oxidation and providing acetyl-CoA to the citric acid cycle. Curiously, up-regulated expression of genes related to lipogenesis, including *MCAT*, *ACC* and *FAS* which are involved in fatty acids synthesis from acetyl-CoA and malonyl-CoA, may be interpreted as a fine-tuning mechanism to build up intramuscular lipid reserves as well as increase their rate of oxidation. Similarly, molecular signs of increased cardiac lipid β-oxidation are reported for rainbow trout in response to exercise [8], [9], sexual maturation [20] and cold acclimation [62], states that also induce cardiac enlargement.

Exercise training had weak effects on the transcription of enzymes involved in glucose metabolism with only *HK* and *PDK* genes being significantly up-regulated by training. Moreover, they metabolically counteract each other, with HK transforming glucose into glucose 6-phosphate (G6P) and favouring glycolysis, while PDK inactivates pyruvate dehydrogenase through phosphorylation, slowing glycolytic formation of acetyl-CoA. Farrell et al. [8] found that training induced elevated levels of enzymes involved in both lipid and glucose oxidation in heart of trained rainbow trout, suggesting an increased dependence on both fuels to cope with the higher demand.

The H-training regime induced cardiac expression of myokines and other inflammation-related factors. Transcriptional regulation of cardiac myokines in response to six weeks of de-training was previously demonstrated in Atlantic salmon [40], with down-regulation of *TNFα*, *IL1β* and *IL6* being associated with higher survival in a viral disease challenge test. Mammalian skeletal muscle is heavily involved in the production and release of myokines, which is further modulated by exercise training. A principal actor is IL6, which is induced by exercise and plays a fundamental role in both metabolic and anti-inflammatory mechanisms [39], [63]. Here, the *IL6* receptor was similarly up-regulated by training. We also found higher cardiac levels of *TNFα* and *COX2*. COX2 is central to production of prostaglandins, inflammatory mediators of lipid origin [64]. *TNFα* and *COX2* elevated expression could also be reflective of an oxidative environment caused by exercise [65]–[67]. The exercise-induced expression of the anti-inflammatory genes *IL1RA* and *TNF decoy receptor* may have occurred in response to the presence of pro-inflammatory molecules as they block the inflammatory effects of IL1β and TNFα, respectively [68]. Thus, the overall net result of myokine activation in the exercised heart may be related to metabolic effects such as muscle growth and lipid oxidation. In light of the present results, as well as others studies showing the production of myokines in the cardiac muscle in response to different stimuli [69]–[73], we suggest that the term “cardiomyokine” should be used when referring to cytokines/myokines that are directly expressed and produced by the cardiac muscle cells. Further research still is needed to establish if these molecules are released to the extracellular space.

In conclusion, we document that aerobic exercise training induces a range of cardiac molecular responses. These are similar to those underlying physiological cardiac growth in mammals, validating our hypothesis of a conserved exercise-induced molecular cardiac response within lower and higher vertebrates. The reported molecular cardiac signature suggests an exercise-induced strengthening of the heart. Similar responses have been seen when cardiac enlargement is induced in fish by other means other than exercise training. We suggest cardiac enlargement involved a mixture of cardiomyocyte enlargement and proliferation. Our gene expression results further imply that this was associated with a more effective contractile machinery, a higher capacity for delivering oxygen to the heart and a greater reliance on lipid oxidation in enlarged mitochondria to satisfy the increased energetic requirements imposed by exercise. Finally, the exercise-induced responses were manifested in an intensity-dependent fashion.

## Materials and Methods

### Experimental Fish

Juvenile Atlantic salmon belonging to the Salmobreed strain were produced and reared at Nofima AS, Sunndalsøra, Norway. Freshwater stage experimental procedures took place on the same research station, which is an approved facility under the Norwegian Animal Research Authority (NARA). Stunning and sampling of fish was done in agreement with the Norwegian regulations. As fish were exposed to different sustainable water velocities that did not induce an un-physiological state, no specific NARA approval was required according to Dr. G. Baeverfjord, member of the national NARA board and local NARA officer at Nofima AS.

### Exercise Training Regimes

**Figure 7:**
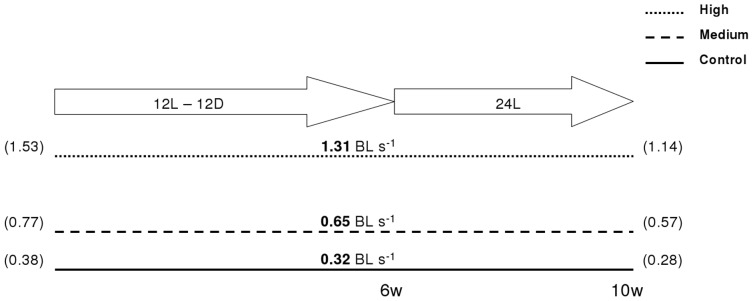
Exercise training experimental design. Atlantic salmon pre-smolts were exercise trained at different relative water speeds (body lengths per second (BL s^−1^)) for ten weeks (w). Throughout the trial, swimming speeds experienced a constant decrease due to fish length increase. Speeds shown in the middle of the figure (bold) are average, while start and end speeds are shown in the left and right, respectively (brackets). The first six weeks took place under a short day-light photoregime (12L-12D), while the last 4 weeks were on a continuous light photoregime (24 L) to induce the smoltification process.

Before the start of the trial, all fish were tagged (Passive Integrated Transponder (PIT), Glass tag Unique 2.12×12 mm, Jojo Automasjon AS, Sola, Norway). After individual measurements of body mass (40.7±0.2 g) and length (15.0±0.3 cm), 77–86 fish were set in each of 9 cylindro-conical tanks (500 l; 82 cm in diameter) and allowed to acclimate for one week under minimum disturbance prior beginning of the experimental trial. The center of each experimental tank was fitted with a plastic pipe (31.5 cm diameter), which reduced the area in the tank with lowest water speed. A frequency-controlled pump (Hanning Elektro Werke, PS 18–300; Oerlinghausen, Germany) directed the water current and a wire mesh fence, attached between the pipe and the edge of the tank, prevented the fish from drifting backwards. The water speeds were calibrated by using the average speed measured at 12 points in the tank (four horizontal locations and three depths at each location (Höntzsch HFA propeller, Waiblingen, Germany with HLOG software). Fish were subjected for 10 weeks to one of three swimming-induced training regimes. Each regime was run in triplicate tanks with constant water speeds of either 5.7 (C), 11.5 (M) and 23 (H) cm s^−1^. At start of the experiment, these speeds were equivalent to 0.38, 0.77 and 1.53 BL s^−1^ for C, M and H, respectively. Due to increases in fish length during the trial, relative water speeds were slightly reduced towards the end of training. Because fish had a similar length across the three groups, this reduction in relative water speed was similar among groups (75% of initial speed in BL s^−1^). Using average fish length during the trial, relative water speeds averaged 0.32 (C), 0.65 (M) and 1.31 (H) BL s^−1^ (Figure 7). To stimulate smoltification, fish were exposed to a short daylight regime (12–12 Light-Dark) the first 6 weeks, followed by continuous light (24 L) the remaining 4 weeks. Tanks were supplied with fresh water and temperature was measured daily (10.5±0.8°C). Oxygen saturation was measured weekly and was maintained over 85% with oxygen supplementation. Measurement of ATPase in gills (n = 10) sampled from each group was conducted in a commercial laboratory, Havbruksinstituttet AS, Bergen, Norway, and confirmed that all fish were sampled within the smolt-window (data not shown).

### Relative Ventricular Mass (RVM)

At the end of the exercise-training period, a total of 30 fish per training regime were killed by a blow to the head; body mass and length were measured, and hearts were dissected out. Ventricles were weighed on an analytical scale after removal of the *atrium*, the *bulbus arteriosus* and any excess blood. RVM was calculated as ventricle weight (g) · [body mass (g)]^−1^ · 100.

### Gene Expression

A total of 44 genes were analyzed for expression levels by quantitative real-time RT-PCR (qPCR). For this, ventricle samples (n = 12/regime) were sampled at the end of the training experiment as previously described, and immediately frozen on liquid nitrogen prior to storage at −80°C until analyzes. Total RNA was extracted from half a ventricle using TRIzol and purified with PureLink RNA Mini Kit (Invitrogen, Carlsbad, CA, USA) including on-column DNA digestion (RNAse-free DNAse set, Qiagen, MD, USA), following manufacturers guidelines and RNA concentration was measured using NanoDrop 1000 spectrophotometer (Thermo Fischer Scientific, Waltham, MA, USA). Synthesis of cDNA was performed on 0.5 µg of total RNA using TaqMan© Reverse Transcription reagents (Applied Biosystems, Foster City, CA, USA) and primed with a mix of oligo dT and random hexamers. Reactions took place on 96 well optical plates on a LightCycler® 480 (Roche Diagnostics, Mainheim, Germany) using 2X SYBR Green Master mix (Roche), 5 µl of cDNA and primer concentrations of 0.42 µm each (final reaction volume was 12 µl). Thermal cycling protocol was as follows: 5 min pre-incubation at 95°C, followed by 45 amplification cycles of 95°C for 10 s, 60°C for 15 s and 72°C for 15 s, followed by a melting curve protocol (95°C for 5 s, continuous increase from 65°C to 97°C) for amplicon specificity assessment. Difference in gene expression ratio (ER) between the groups was assessed by the method described by Pfaffl et al. [74], normalized using an averaged value from two genes, *elongation factor 1α* and *18S rRNA*. PCR efficiency was assessed by six 10-fold serial dilutions of pooled sample templates for each primer pair. The cDNA was diluted 10-fold before use for all the genes, except for the abundantly expressed *18S*, which was diluted 1000-fold. All primers were designed using ePrimer3 from the EMBOSS online package [75] and synthesized by Invitrogen (Table S1).

### Mitochondrial Quantification

The ratio of mitochondrial DNA to genomic DNA (mtDNA/gDNA) was calculated for the C and H regimes by qPCR, as an estimate of the number of mitochondria per cardiac cell. The same individual fish used for the gene expression study were analyzed (n = 12/regime). Total DNA was isolated using Dneasy kit (Qiagen) according to manufacturer instructions and diluted 200-fold before use. Concentration was measured on NanoDrop 1000 spectrophotometer (Thermo Fischer Scientific) and quality was checked on a 1% agarose gel. The relative difference in the abundance of mitochondria per cell was calculated after qPCR amplification of specific mitochondrial (D-loop) and nuclear (*myoD*) DNA sequences. For the latter, primers spanned an intron-exon region (Table S1). Comparisons among groups, as well as the amplification reactions and primer efficiency calculations were performed similarly to the gene expression analyses described above, but using *myoD* as reference gene against which the levels of D-loop were normalized and quantified.

### Immunofluorescence and Mitochondrial Staining

Hearts sampled for immunofluorescence (IF) were embedded in paraffin and 7 µm sections were prepared. After paraffin removal and dehydration, microwave facilitated antigen retrieval was carried out for 20 min in 10 mM Tris-Hcl pH 10. After rinsing in PBST (phosphate buffered saline with 0.02% Tween 20), the tissue sections were permeabilized for 20 min in 1×PBST with 1% Triton X100. Blocking was carried out for 2 h in 1× PBST with 5% skimmed dry milk. Polyclonal TNFα (100-fold dilution; obtained according to the method described in Bethke et al. [76] and provided by L. Mercado at Pontificia Universidad Catolica de Valparaiso, Chile) PCNA (70-fold dilution; PMID 17349083; Zymed Laboratories Inc., CA, USA) and VEGF (50-fold dilution; PMID 15177948; Santa Cruz Biotechnology, CA, USA) primary antibodies were diluted in 1×PBST with 2% dry milk and 0.01% Triton X 100 and applied to the sections over night at 4°C. After extensive washing in 1×PBST, the sections were incubated for 2 h with Alexa conjugated secondary antibodies diluted 200-fold (Invitrogen, Carlsbad, CA, USA). Finally, the sections were mounted after several washes in 1×PBST and nuclear staining with DAPI. All images were captured using a Zeiss Axioplan Z1 microscope and post processed using the Zeiss Axiovison software. Identical exposure and image manipulation settings were applied to the images to enable comparison between swimming regimes. For visualization of TNFα, image stacks were deconvolved using an iterative algorithm in the Axiovision software.

Staining of cardiolipin in the mitochondrial membranes was carried out using 10-nonyl acridine orange (NAO; PMID 16172211). Briefly, paraffin was removed from 3 µm sections before rehydration and permeabilization with 1% Triton X100 in 1×PBST. Quenching of auto fluorescence was achieved by incubating the sections for 2 min in 0.1% Sudan black dissolved in 70% ethanol. After washing in 1×PBST, the sections were incubated for 10 min in 10 µM NAO diluted in 1×PBST, before washing and mounting. A total of 17700 and 15600 mitochondria from the control (n = 5) and exercised fish (n = 6), respectively, were analyzed for fluorescence intensity and size using a semiautomatic script which isolated the mitochondria from the background using fluorescence intensity and size segmentation.

### Statistics

Statistical analyses among the different groups were assessed by analysis of variance (ANOVA) and comparison between groups with Tukey’s HSD post-hoc test. Student’s *t*-test was performed between C and H groups for those analyses on which only these two groups were of interest (*a priori* comparisons for mtDNA/gDNA ratio, mitochondrial area, immunofluorescence and RVM). We have further used Student’s *t*-test between C and H groups for those highly interesting genes on which p-value after ANOVA was close to significance (0.05<p<0.08). Correlation between training intensity, RVM and gene expression was assessed by Pearsons’*r* (SAS 9.1; SAS Institute Inc., NC, USA). Differences and correlations were considered significant at p<0.05.

## Supporting Information

Table S1 Genes and primer sequences used for the qPCR analyzes.
(DOC)
