# Supplementary material for: Cardiac Molecular-Acclimation Mechanisms in Response to Swimming-Induced Exercise in Atlantic Salmon
Source: PLoS One. 2013 Jan 25;8(1):e55056. doi: 10.1371/journal.pone.0055056 (PMC3555865; doi:10.1371/journal.pone.0055056)
Supplement: Table S1 — Genes and primer sequences used for the qPCR analyzes. (DOC) [file pone.0055056.s001.doc]

**Table S1**. Genes and primer sequences used for the qPCR analyzes.

| **Genes** | **Short Name** | **Sequence 5' to 3'** | **Accession number** |
| --- | --- | --- | --- |
| myocyte-specific enhancer factor 2C | MEF2C | F-CACCGTAACTCGCCTGGTCT | GU252207 |
|  |  | R-GCTTGCGGTTGCTGTTCATA |  |
| NK2 homeobox 5 | NKX2.5 | F-CCCAGTACGTCCACACCCTT | DW550500 |
|  |  | R-GGAGGTCGGTAAGGCACAGT |  |
| GATA binding protein 4 | GATA4 | F-TCTCCATTCGACAGCTCCGT | HM475152 |
|  |  | R-CATCGCTCCACAGTTCACACA |  |
| actin | ACTA1 | F-CACAAACTGGGATGACATGG | EG835630 |
|  |  | R-GTTGGCTTTGGGATTGAGTG |  |
| troponin T2 | TNNT2 | F-GCTGGAGGCTGAGAAGTTTG | CK882307 |
|  |  | R-TCAGGCCTCTCTTGGTTCTC |  |
| Ca^2+^ channel, voltage-dependent, L type, α1D subunit | DHPRa1D | F-TAGCGCTGATAGACGGGACT | EG648547 |
|  |  | R-CATGCCAACATCACTTCCTG |  |
| ryanodine receptor isoform 1 | RYR1 | F-CTCTACCGGGTGGTCTTTGA | DW541352 |
|  |  | R-ACCTGCTCTTGTTGGTCTCG |  |
| FK506 binding protein 1b | FKBP1B | F-CAGGTATGCTGCAAAATGGA | DY706243 |
|  |  | R-CATCTGTGCGATTCCTTCCT |  |
| calsequestrin-1 | CALSEQ1 | F-ACAGCTGAGGAACTGGAGGA | BT045346 |
|  |  | R-GTGCCTATCCTCCCTGTCAA |  |
| sarco-endoplasmic reticulum Ca^++^ ATPase 2 | SERCA2 | F-AGTCCTCTGCGTTTGATGGT | Ssa#TC111867 |
|  |  | R-GTCTCTGTAGCCTCGCCAAC |  |
| erythropoietin | EPO | F-CATCTAACCAGACTGACGTGCT | DQ288854 |
|  |  | R-CACTTGTCTGATGCTGGCAA |  |
| erythropoietin receptor | EPOR | F-TCTCGATCAGATTTGGGCTC | BT045834 |
|  |  | R-CTCGAGCTCATCGGACTGTAAT |  |
| vascular endothelial growth factor | VEGF | F-AGACAGCCCACATACCCAAG | NM_001124417 |
|  |  | R-GAAGACGTCCACCAGCATCT |  |
| vascular endothelial growth factor receptor 2 | VEGF-R2 | F-GTTGCAAAAGGCATGGAGTT | AJ717303 |
|  |  | R-TCTCTGGCCAGTCCAAAATC |  |
| inducible nitric oxide synthase | iNOS | F-GCTAAACTGTGCCTTCAACTCCA | AF088999 |
|  |  | R-CTCCATTCCCAAAGGTGCTAGTTA |  |
| peroxisome proliferator-activated receptor α | PPARa | F-TCCTGGTGGCCTACGGATC | DQ294237 |
|  |  | R-CGTTGAATTTCATGGCGAACT |  |
| peroxisome proliferator-activated receptor β | PPARb | F-GAGACGGTCAGGGAGCTCAC | AJ416953 |
|  |  | R-CCAGCAACCCGTCCTTGTT |  |
| PPARγ cofactor 1α | PGC1a | F-GTCAATATGGCAACGAGGCTTC | FJ710605 |
|  |  | R-TCGAATGAAGGCAATCCGTC |  |
| acyl-CoA oxidase | ACO | F-CCTTCATTGTACCTCTCCGCA | DQ364432 |
|  |  | R-CATTTCAACCTCATCAAAGCCAA |  |
| carnitine palmitoyltransferase 1 | CPT1 | F-TCCCACATCATCCCCTTCAACT | AM230810 |
|  |  | R-TGTCCCTGAAGTGAGCCAGCT |  |
| lipoprotein lipase | LPL | F-TGCTGGTAGCGGAGAAAGACAT | BI468076 |
|  |  | R-CTGACCACCAGGAAGACACCAT |  |
| malonyl-CoA decarboxylase | MCD | F-TGCTCGACACTCAACAAAGG | DW540094 |
|  |  | R-ACTCCCGATTCCACCCTACT |  |
| malonyl CoA-acyl carrier protein transacylase | MCAT | F-TGCTGCAGGATTTAGTGTGG | BT059434 |
|  |  | R-AGCTCTGACGCTTTCTGCAT |  |
| acetyl-CoA carboxylase | ACC | F-GGATTGCCTGTATCTTGGAC | DW573070 |
|  |  | R-CTGGACGATACTCTGAGTGTTC |  |
| fatty acid synthase | FAS | F-TGCCTCAGCACCCTACTCTG | BT060359 |
|  |  | R-GCTTTACAACCTCAGGATTGGC |  |
| glucose transporter type 4 | GLUT4 | F-GGCGATCGTCACAGGGATTC | AF247395 |
|  |  | R-AGCCTCCTCAAGCCGCTCTT |  |
| hexokinase II | HK | F-GGGAGATAGTGAGGAACGTACT | DY720410 |
|  |  | R-GGGAGATAGTGAGGAACGTACT |  |
| phosphofructokinase | PFK | F-AATCCATCGGCGTTCTGACAAGC | NM_001173694 |
|  |  | R-GCCCGTACAGCAGCATTCATACCTT |  |
| pyruvate kinase | PKM2 | F-GAAGGGAGCTCACATCAAGC | EL697804 |
|  |  | R-TAGACGTGACCTCCGACCTT |  |
| pyruvate dehydrogenase E1 component subunit b | PDHB | F-CATGCCCTACGCTAAGATCC | BT045854 |
|  |  | R-AGGAGTGGGGAAGGAAACAT |  |
| pyruvate dehydrogenase kinase 3 | PDK3 | F-CCTCAGGAAGATCGACAAGC | NM_001139694 |
|  |  | R-GAAATGGGGAGACCATACCC |  |
| phosphoenolpyruvate carboxykinase | PEPCK | F-AGGGCATGGACCAGGAACTCC | BT072418 |
|  |  | R-GGGCTCTCCATCCTGGGATGT |  |
| glycogen phosphorylase | PYGM | F-AAGGCCACTCTGTTCAAGGA | BT058854 |
|  |  | R-TGCAATGACTTCAGCCAGTC |  |
| tumor necrosis factor α | TNFa | F-AGGTTGGCTATGGAGGCTGT | NM_001123589 |
|  |  | R-TCTGCTTCAATGTATGGTGGG |  |
| interleukin 1 β | IL1b | F-GTATCCCATCACCCCATCAC | NM_001124347 |
|  |  | R-TTGAGCAGGTCCTTGTCCTT |  |
| interleukin 6 | IL6 | F-ATGAAGGTTGCTGAGGTAGTGG | NM_001124657 |
|  |  | R-TAGCAGTGTTGTCATGGTTACTGG |  |
| cyclooxygenase 2 | COX2 | F-CTATGTCCTGACATCTCGCTCACAT | AY848944 |
|  |  | R-TCCTGCGGTTCCCATAGGT |  |
| 5-lipoxygenase-activating protein | FLAP | F-TCTGAGTCATGCTGTCCGTAGTGGT | CA369467 |
|  |  | R-CCTCCCTCTCTACCTTCGTTGCAAA |  |
| nuclear factor κB p105 subunit 1 | NFkBp105 | F-CAGCGTCCTACCAGGCTAAAGAGAT | CA341859 |
|  |  | R-GCTGTTCGATCCATCCGCACTAT |  |
| interleukin 6 receptor subunit α | IL6Rsa | F-GTGGCAGTTCCTCCAGAGAG | NM_001173710 |
|  |  | R-GGAGGTAGCACTGAGGGACA |  |
| nuclear factor κB inhibitor alpha-1 | NFkB inhibitor | F-TGGTAACCTTGTGAAGGAGCTGGA | BT125324 |
|  |  | R-GCTCAGCATGTTCTGTGGCTTCAT |  |
| interleukin 1 receptor antagonist | IL1RA | F-TCCCTGTGGTCCTCAACTTC | EF579740 |
|  |  | R-GCCTGGATCTCCTCATCATC |  |
| TNF decoy receptor | TNF decoy-R | F-CAGAAGTTTTCTGTGTGTGCCC | EG881931 |
|  |  | R-AACCAGTCCTACATGCTGAGCA |  |
| interleukin 15 | IL15 | F-TTGGTTTTTGCCCTAACTGC | EG792923 |
|  |  | R-CAGGTCCATCGCACTCTTTT |  |
| elongation factor 1α | EF1a | F-CACCACGGGCCATCTGATCTACAA | BT072490 |
|  |  | R-TCAGCAGCCTCCTTCTCGAACTTC |  |
| 18S | 18S | F-GCCCTATCAACTTTCGATGGTAC | AJ427629 |
|  |  | R-TTTGGATGTGGTAGCCGTTTCTC |  |
| mt D Loop B (mtDNA) | D Loop B | F-CCCCTGAAAGCCGAATGTAA | NC001960 |
|  |  | R-CGACCTTGTTAGACTTCTTTGCTTG |  |
| myogenic differentiation 2 (gDNA) | MyoD2 | F-CAGAGCCAGGATTACACTCGTTACA | AJ557150 |
|  |  | R-GCATGTCGCTGGTGTTGAAG |  |
|  |  |  |  |
|  |  |  |  |
